# Correction: Sandlersky et al. Multispectral Remote Sensing Data Application in Modelling Non-Extensive Tsallis Thermodynamics for Mountain Forests in Northern Mongolia. *Entropy* 2023, *25*, 1653

**DOI:** 10.3390/e26060523

**Published:** 2024-06-18

**Authors:** Robert Sandlersky, Nataliya Petrzhik, Tushigma Jargalsaikhan, Ivan Shironiya

**Affiliations:** 1 V.N. Sukachev Laboratory of Biogeocenology, A.N. Severtsov Institute of Ecology and Evolution of the Russian Academy of Sciences, Leninsky Prospect 33, 119071 Moscow, Russia; petrzhik.nat@mail.ru (N.P.); ivan.shironya@gmail.com (I.S.); 2 International Laboratory of Landscape Ecology, National Research University Higher School of Economics, Pokrovskiy Bulvar 11, 109028 Moscow, Russia; 3 Laboratory of Forest Phytocoenology, Botanic Garden and Research Institute Mongolian Academy of Sciences, Peace Avenue 54a, Bayanzurkh District, Ulaanbaatar 13330, Mongolia; tushigmaaj@mas.ac.mn

There were some errors in the original publication [1], the term “cedar” was inaccurately used instead of “pine”, and a battery of corrections have been made throughout the main text.

There was also a spelling error in the name of the study area, the coordinates were displayed in an incorrect format. Corrections have been made to the following paragraphs, respectively.

Section 2.1, Paragraph 1:

The study area is situated within the Horidol-Saridag Strictly Protected Area (50.90 N, 99.88 E), along the ridge that separates Lake Khövsgöl and the Darkhad Valley (Figure 1). This region falls under the Ulaan Taiga Specially Protected Areas designation, including three additional areas: Ulaan Taiga Strictly Protected Area and Tengis–Shishged National Park [56]. These regions are located in the northernmost aimag (province) of Khövsgöl in Mongolia. Elevations within this study area range from 1600 m in the Darkhad depression to 3000 m above sea level at the Khuern Uul mountain. The predominant geological composition of this region consists of limestone carbonates from the Cambrian period. Geologically, the area underwent at least two glaciations during the late Pleistocene [57].

Section 4, Paragraph 1:

Let us compare our results with the results of other boreal forests, namely with the results of a similar analysis of the boreal ecosystems of the European plain (56.30 N, 32.53 E Central Forest Reserve, CFR) [52] and the boreal forests of the north-eastern Baikal region (55.35 N, 109.81 E Baikal region, BR) [72]. These ecosystems form a gradient along the absolute height and degree of continentality. The CFR has an altitude of 250 m above sea level, and has a temperate continental climate, flat topography, and, therefore, excess moisture most of the year, while the BR is located in an area of sharp continental climate, moderated by the influence of Lake Baikal in the summer, with elevations of 480–1240 m above sea level, compared to 1600–3000 m above sea level in Horidol Saridag (HS). The areas of the analyzed regions are comparable. It is also worth noting that the calculations for BR were carried out within the framework of the BGS model, without estimating the q-index, while calculations for CFR were carried out in two systems [53,73], and it was shown that the relationship between spatiotemporal variation in energy balance variables is generally similar between NSM and BGC.

Additionally, “2 m” was mistakenly printed instead of “20 m” in the original publication [1]. A correction has been made to Section 2.3, Paragraph 4:

The impact of terrain on energy conversion was assessed by employing multiple regression analysis on the invariants obtained from morphometric characteristics. Field measurements of ecosystem properties were conducted using the line transect method. A 1-kilometer-long transect with control sites every 20 m was established along the steep southern slope, ranging from 2000 to 2380 m asl (Figure 2). Comprehensive descriptions of the soil and vegetation were recorded for almost all sites. Field characteristics, including stand basal area and canopy percentage projection to calculate the leaf area index (LAI), were estimated. Furthermore, fresh phytomass was sampled from a 25 × 25 cm surface square at each site in triplicate, and the dry phytomass weight was determined. The transect spanned pine-dominated forest communities at the base and middle parts of the slope (2000–2200 m asl) and extended to alpine meadows (2200–2350 m asl) before transitioning into barren rocky peaks at higher altitudes.

Lastly, “SBA” was mistakenly printed instead of “BSA” in the original publication [1]. A correction has been made to Section 3.3, Paragraph 3:

While the correlation between ecosystem properties and order parameters is comparatively weaker than the correlation of individual variables with ecosystem properties, their interaction pattern remains consistent. Notably, wood vegetation mass enhances solar energy absorption, exergy, and productivity, while diminishing bound energy. Moreover, the correlation between residuals and solar energy absorption values surpasses that between residuals and field data in general. Field data correlations with their respective values are stronger than those between model residuals and other parameters. These intricate relationships between order parameters and key measured vegetation properties, such as BSA and stand canopy density, are graphically represented in Figure 5.

The authors state that the scientific conclusions are unaffected. This correction was approved by the Academic Editor. The original publication [1] has also been updated.
